# The Prevalence and Associated Factors of Neck Pain among Ministry of Health Office Workers in Saudi Arabia: A Cross Sectional Study

**DOI:** 10.3390/healthcare10071320

**Published:** 2022-07-16

**Authors:** Anas Mohammed Alhakami, Adel Madkhli, Mohammed Ghareeb, Abdulaziz Faqih, Ismail Abu-Shamla, Tariq Batt, Fatemah Refaei, Ahmad Sahely, Bassam Qassim, Ayman M. Shami, Abdulaziz H. Alhazmi

**Affiliations:** 1 King Faisal Medical City for Southern Region, Medical Rehabilitation Center, Abha 62523, Saudi Arabia; alhakami.anas@gmail.com; 2 Physiotherapy Department, Abu-Arish General Hospital, Jazan 45142, Saudi Arabia; amadkhli@moh.gov.sa (A.M.); mnghareeb@moh.gov.sa (M.G.); faqih.abdulaziz10@gmail.com (A.F.); iabushamla@moh.gov.sa (I.A.-S.); tbatt@moh.gov.sa (T.B.); 3 Jazan University Hospital, Pharmacy Department, Jazan University, Jazan 45142, Saudi Arabia; frefaei@jazanu.edu.sa; 4 Faculty of the Applied Medical Sciences, Jazan University, Jazan 45142, Saudi Arabia; sahely327@hotmail.com; 5 Respiratory Care Department, King Fahd Central Hospital, Jazan 45142, Saudi Arabia; b.y.g@hotmail.com; 6 Faculty of Medicine, Jazan University, Jazan 45142, Saudi Arabia; aymnshamy97@gmail.com

**Keywords:** neck pain, Saudi Arabia, healthcare workers, Ministry of Health

## Abstract

(1) Background: Neck pain is the most common type of musculoskeletal problem affecting office workers. Various occupational risk factors have been linked to neck pain. This study aims to assess the prevalence and risk factors of neck pain among office workers at the Ministry of Health in Saudi Arabia. (2) Methods: A cross-sectional study was conducted, and the participants completed an online questionnaire based on the Standardized Nordic questionnaire and Quality of Life Scale Brief Version to evaluate their neck pain and the physical, psychological, social, and environmental factors that might affect their conditions. A descriptive analysis was conducted for the data and a logistic regression was performed to ascertain the effects of biodemographic and occupational factors on the likelihood of having neck pain. (3) Results: A total of 413 subjects (176 females and 237 males) participated in our study with an average age of 33.6 ± 8 years. The prevalence of neck pain in our participants was 64% during a twelve-month period. Females were less likely to suffer neck pain than males (OR = 0.52, 95%CI [0.30,0.87]), and age, BMI, level of education, and profession were not associated with likelihood of having neck pain. However, reduced working hours were associated with a reduction in the likelihood of having neck pain (OR = 0.42, 95%CI [0.33,0.53]). (4) Conclusion: Neck pain affects a large proportion of the office workers at the Ministry of Health, and this pain is significantly associated with long working hours and males. Thus, there is a need for future research that can investigate how associated factors can be managed to reduce the long-term impact of neck pain on workers’ lives. Quality improvement approaches might be used to implement effective interventions for the prevention and management of work-related risk factors that can cause neck pain.

## 1. Introduction

Musculoskeletal disorders (MSDs) are considered the second highest contributor to global disability [1]. MSDs affect a significant proportion of workers, especially those whose work requires a significant amount of physical and psychological effort. The neck pain associated with MSDs is found as a major cause of morbidity and disability in everyday life and at work, affecting around 34.4% of office workers globally every year [1,2,3].

Neck pain is a complex problem and might be associated with several factors, such as an individual’s functional level, physical activity, psychosocial status, and work habits [4]. Moreover, the prevalence of neck pain has been increasingly reported in Asia more than in Western countries which suggests the influence of other factors, such as lifestyle and ethnicity [5]. The burden of neck pain has been increasing especially in the low-income and middle-income countries, negatively affecting individuals’ quality of life and increasing the pressure on healthcare systems [1].

Healthcare workers might be at risk of experiencing neck pain at some points in their careers. A systematic review of work-related MSDs found that 35–45% of doctors, nurses, and midwives suffer from neck, shoulder, and upper back pain [6]. In another study, the prevalence was 50–93% among dental personnel, with the shoulder being the most frequently affected area [7]. Neck pain outcomes in this population might lead to a reduction in working hours, limitation of daily activities, sleep disruption, or attrition from the workforce at worst [6,7,8].

The research in this area has been growing to explore the most effective interventions that can help people with neck pain and reduce their need for surgical interventions. Various interventions have been studied, such as active breaks and postural shifts, which are reported to be effective methods to reduce neck pain among office workers [9]. As the proportion of people at risk of neck pain is expected to increase in the next decade [1], appropriate care plans are needed for the management and prevention of neck pain. To gain better knowledge about this problem, there is a need for a better understanding of the cause-and-effect relationship between neck pain and risk factors that might affect the individual in a certain context. The current study aims to determine the prevalence of neck pain and associated factors among office workers who are working in the Ministry of Health, Saudi Arabia.

## 2. Methods

### 2.1. Participants

In this cross-sectional study, participants were selected by convenient sampling, and an online questionnaire was sent to the official emails of Ministry of Health workers using the Google Form platform between 1 November 2021 and 31 January 2022. Only investigators had the access to participants’ responses, and frequent verification of the participants’ responses was conducted. Inclusion criteria were people aged over 18 years, and full-time Ministry of Health office workers with any type or severity of neck pain. Participants were excluded if they were pregnant females, had a recent infection, were admitted to hospital for any other health conditions, or workers with low back pain. 

### 2.2. Questionnaire

A self-administered online questionnaire was developed based on published pretested questionnaires [10,11,12]. The questionnaire is divided into three parts: the first part is about socio-demographic characteristics, such as age, gender, height, weight, nationality, education level, marital status, smoking status, profession, and working duty. In the second part, neck pain was assessed by the question “Have you ever had neck trouble (ache, pain, or discomfort)?” The subjects who answered “yes”, completed the Standardized Nordic questionnaire [10,11] and completed the third part which was the Quality of Life Scale Brief Version (WHOQOL-BREF) questionnaire [12] to evaluate four domains: physical, psychological, social, and environmental characteristics of participants with neck pain.

### 2.3. Sample Size

The sample size of this study was calculated using a statistical formula for a cross-sectional survey design. The anticipated population proportion (p) of the sample is estimated to be 50% because it is the safest choice for (p) since the sample size required is largest when *p* = 50%. No previous study was conducted in the region dealing with musculoskeletal pain among Ministry of Health office workers. Other parameters for sample-size calculation included a 95% confidence interval (CI), a marginal error of 5%, and a nonresponse rate of 20%. The final sample size was 384 Ministry of Health office workers; however, 413 participants were recruited in this study to increase the significant power of this study.

### 2.4. Ethical Approval

The study was approved by Jazan Health Ethics Committee, Ministry of Health, Saudi Arabia, with approval number (#2133, dated 26 April 2021). This study was conducted following the ethical guidelines of the Helsinki Declaration and the local guidelines of the National Committee of Bioethics, Saudi Arabia. All participants were asked for their willingness to take part in the study and the objectives of the study were thoroughly explained to them at the beginning of the survey. Collected data were kept confidential and used only for the purpose of research within the objectives of this research. Additionally, the questionnaires did not include participants’ personal data or any other methods of identification. Participants of this study were given the right to continue or withdraw at any time from the study.

### 2.5. Data Analysis

Data were tabulated and descriptive statistics were conducted, including means and frequency tables which were prepared using IBM SPSS v.26 (IBM Corp., Armonk, NY, USA). Logistic regression was performed to ascertain the effects of age, gender, BMI, level of education, profession, and working hours on the likelihood of having neck pain during last twelve months.

## 3. Results

A total of 413 subjects participated in our study with 176 (42.6%) females and 237 (57.4%) males (Table 1). The majority (87.2%) of participants were Saudi with an average age of 33.6 ± 8 years (30.5 ± 6.1 years for females and 35.9 ± 8.3 years for males). The participants had an average Body Mass Index (BMI) of 25.7 ± 5.2 (24.8 ± 5.7 for females and 26.4 ± 4.8 for males).

Based on their height and weight, the participants were further categorized based on their Body Mass Index (BMI) into underweight (6.5%), normal (41.4%), overweight (32%), or obese (20%). The majority of the participants were married (68.3%), around 28.6% of them were unmarried, 2.9% of the participants were divorced, and 0.2% were widowed. About 22% of the participants informed that they smoke. In terms of their educational level, the study included participants with different levels which included; Secondary School (7.3%), Diploma (29.8%), Bachelor’s (50.4%), Master’s (9%), and Doctorate (3.6%). 

Out of the 413 participants 4.6% were technicians, 26.6% were nurses, 23.7% were specialists, 17.7% were physicians, 7% were senior specialists, 2.9% were pharmacists, 1.2% were consultants, 0.2% financial analysts, 1.7% were engineers, 18.4% were administrative assistants, 0.5% were receptionists, 1% were interns, and 0.5% were security personnel. Regarding daily working hours, 24% of the participants indicated that they work for ≤7 h, 60% work for 8 h, and 16% work for more than 8 h every day. 

### 3.1. Prevalence of Neck Pain

The 12-month prevalence of neck pain was 63.9% and about 51.6% of participants reported that they had experienced neck pain in the previous seven days.

Based on 264 responses from 413 participants who reported neck pain, the duration varied during the previous year. Of these, 41.8% reported pain lasting 1–7 days, 7.2% reported pain lasting 8–30 days, 10.3% reported having pain that lasted more than 30 days and 6.15% reported everyday pain during the previous year.

When asked whether neck pain had an impact on their job activity during the previous year, 74% responded affirmatively, with 60.2% acknowledging that it had an impact on their daily activities.

In response to the question about the length of time the neck pain prevents them from conducting their normal work, 41.8% of respondents said 1–7 days, while 34.6% of the respondents suffering from neck pain said 0 days.

When asked how long the neck pain had kept them from completing their typical jobs, 41.8% of individuals with neck pain indicated they had been unable to work for 1–7 days, whereas 34.6% of those who had neck pain said it did not affect their work.

Out of 262 respondents having neck pain in the last 12 months, 30.5% of them have consulted a physician or a physical therapist (Table 2).

In response to the question about neck pain affecting their concentration, only 1.2% said that neck pain doesn’t affect their concentration, 13.1% told that it’s affected to a minimum effect, 42.1% participants said it had a moderate effect on concentration, 33.3% of participants said the neck pain greatly affect their concentration, and 10.3% of participants’ concentration was extremely affected. The neck pain sometimes caused stress and anxious feelings among 11.9% of participants, frequently caused anxiety and depression among 45.6% of participants, and always caused anxiety and depression among 28.6% (Table 3).

### 3.2. Factors Affecting the Neck Pain

Logistic regression was performed to ascertain the effects of age, gender, level of education, profession, and working hours on the likelihood of having neck pain during the last twelve months. The logistic regression model was statistically significant, X2 (6) = 132.794, *p* = 0.0001. The model explained that 37.7% (Nagelkerke R^2^) of the variance in neck pain correctly classified 89.4% of cases. Females were slightly less likely to suffer from neck pain (OR = 0.52, 95%CI [0.30, 0.87]). The age, BMI, level of education, and profession were not associated with likelihood of having neck pain, but decreased working hours were associated with a decrease in the likelihood of having neck pain (OR = 0.42, 95%CI [0.33, 0.53]).

## 4. Discussion

This study is one of the few to explore the prevalence and associated factors of neck pain in Saudi Arabian healthcare workers. The findings of this study confirm that 63.9% of the office workers at the Ministry of Health in Saudi Arabia commonly suffer from neck pain in a 12-month period, with 51.6% of them experiencing pain in the week prior to participation. This result is close to the findings of other studies conducted in Canada and Australia where the prevalence of neck pain for people who are working in healthcare services was 66.7% and 60.9%, respectively [13,14]. Compared to other populations, the prevalence of neck pain in healthcare workers was higher than the prevalence in office workers in other disciplines (54.5%) [15], Korean community residents (20.8%) [16], and desk-job workers (34.4%) [3]. Moreover, the 7-day prevalence was higher than those reported among young Brazilian people (36%) [17], undergraduate students in Hong Kong 17.5% [18], and the general British population 24% [19]. Although the findings from different studies confirm a high prevalence of neck pain, the ratio of risk factors can differ based on the context of each study.

Jobs with more sitting time seem to increase the risk of neck pain: for instance, the lifetime prevalence of neck pain in another study in Saudi Arabia was higher (84.6%) among dentists than other office workers in the Ministry of Health [20]. However, the variation in prevalence between different studies may be related to differences in socioeconomic information and data collection methods. The high prevalence of neck pain reported in this study seems to impact participants’ jobs. For example, neck pain reduces the participants’ working and daily activity, and their concentration during working hours. Moreover, 41.8% cannot complete their work for a period of 1–7 days. These results are in agreement with the findings of many other studies around the world [21,22,23].

It is been reported that job dissatisfaction and physical and mental tiredness can increase the risk of neck pain, which is also in parallel with our findings [2,22]. Psychological disorders have shown a significant association with neck pain in some studies [18,24]. In this study, 89.1% of the participants reported that neck pain caused negative feelings that can lead to psychological disorders, such as depression and anxiety. As there is a consensus that anxiety and depression can promote pain sensation, it might be suggested to investigate the dual relationship between neck pain and depression and anxiety [25].

Our study reveals that the sex of the participants and duration of working hours are factors that are associated with the risk of neck pain (Table 4). These results are similar to another study that examined the prevalence of neck pain and associated factors among military office workers [22], and similar to a British study that reported the prevalence and associated occupational factors of neck pain [19]. However, the impact of gender needs further investigation as other studies found a higher prevalence in female participants.

Despite being one of the few studies in Saudi Arabia that report on the prevalence of neck pain, this study bears some limitations. As it is a cross-sectional study, there might be limited time to cover all possible causes and other effect factors, and future research can examine the factors associated with neck pain. Further, the study uses a subjective method for data collection (self-administered questionnaire) and participants’ responses in this method might be affected by their psychological and emotional status which might affect the conclusion of the study. Lastly, the generalizability of the study’s findings might be limited because of the population which was purposefully selected from one setting and within a small range of ages. Thus, further national research should be conducted to evaluate other factors associated with neck pain and tailored educational programs could be developed to minimize neck-pain risk factors among office workers. 

## 5. Conclusions

Office workers at the Ministry of Health are highly affected by neck pain. It seems that neck pain is linked to longer working hours and the male sex. Thus, to obtain a better understanding of the problem, future studies might include a larger population from various workforce contexts, and with different demographic characteristics. 

## Figures and Tables

**Table 1:** Demographic characteristics of the participants (Total number = 413).

**Variable**	n	%
Gender		
Male	237	68.3
Female	176	28.6
**Age**		
Male (mean ± SD)	35.9 ± 8.3 years	
Female (mean ± SD)	30.5 ± 6.1 years	
**Marital Status**		
Married	282	68.3
Single	118	28.6
Divorced	12	2.9
Widowed	1	0.2
**BMI**		
Underweight	27	6.5
Normal	171	41.4
Overweight	132	32.0
Obese	83	20.1
**Nationality**		
Saudi	360	87.2
Non-Saudi	53	12.8
**Education level**		
Secondary school	30	7.3
Diploma	123	29.8
Bachelor’s	208	50.4
Master’s	37	9.0
Doctorate	15	3.5
**Smoking**		
Yes	90	21.8
No	323	78.2
**Profession**		
Technician	19	4.6
Nurse	110	26.6
Specialist	98	23.7
Physician	73	17.7
Senior Specialist	3	0.7
Pharmacist	12	3.0
Consultant	5	1.2
Financial Analyst	1	0.2
Engineer	7	1.7
Administrative Assistant	76	18.4
Receptionist	2	0.5
Intern	4	1.0
Physician Assistant	1	0.2
Security	2	0.5
**Working Hours**		
<4 h	20	5.0
5–7 h	79	19.0
8 h	247	60.0
>9 h	67	16.0

**Table 2:** Neck pain history and its effect on work and daily activities.

Question	Gender	Yes (%)	No (%)
Have you had neck trouble (ache, pain or discomfort) in last twelve months?	Male	57.8	42.2
	Female	72.2	27.8
2. Has neck trouble caused you to reduce your working activity during the last 12 months?	Male	70.1	29.9
	Female	78	22
3. Has neck trouble caused you to reduce your leisure activity during the last 12 months?	Male	61.3	38.7
	Female	59.1	40.9
4. Have you had neck trouble at any time during the last 7 days?	Male	40.5	59.5
	Female	63.2	36.8
5. Have you been seen by a doctor or physiotherapist because of neck trouble during the last 12 months?	Male	30.1	69.9
	Female	31	69

**Table 3:** Negative consequences of Neck pain.

Questions	Gender	Never	Rarely	Sometimes	Most of the Time	Always
How often do you have negative feelings such as stress and anxious feelings?	Male (%)	3	5.3	12.1	44	35.6
	Female (%)	10	10	11.7	47.5	20.8
How well are you able to concentrate?	Male (%)	1.5	8.3	39.4	37.9	12.9
	Female (%)	1	18.2	45	28.3	7.5
How would you rate its effect on your quality of life?	Male (%)	1.5	5.3	18	49.6	25.6
	Female (%)	2	5	26	49.6	17.4

**Table 4:** Regression analysis to ascertain the effects of age, gender, level of education, profession, and working hours on the likelihood of having neck pain during last twelve months ^&^.

	*p*-Value	aOR	95% C.I.	
			Lower	Upper
Age	0.373	0.985	0.953	1.018
Gender (Reference: Female)	0.014 *	0.515	0.303	0.873
Body Mass index	0.125	1.038	0.990	1.089
Education Level	0.398	0.885	0.668	1.174
Profession	0.925	1.004	0.931	1.082
Working hours	0.0001 *	0.417	0.332	0.525

aOR: adjusted odds ratio. CI: confidence interval. * The alpha criterion for *p*-value was set to 0.05. ^&^ Binary logistic regression was used for multivariate analysis using the factors associated with absence of neck pain, such as age, gender, BMI, education level, profession, and working hours.

## Data Availability

Data are available upon a reasonable request from corresponding author.
